# Bridging evidence and practice: multi-method evaluation of implementation fidelity of a multilevel hypertension intervention in a federally qualified health center

**DOI:** 10.3389/frhs.2026.1856884

**Published:** 2026-07-17

**Authors:** Antoinette Schoenthaler, Franzenith De La Calle, Nordine D’aguilar, Elaine De Leon, Jacalyn Nay, Doreen Collela, Isaac Dapkins, Soumik Mandal, Milagros C. Rosal

**Affiliations:** 1 Institute for Excellence in Health Equity, NYU Langone Health, New York, NY, United States; 2 Division of General Internal Medicine and Clinical Innovation, NYU Grossman School of Medicine, New York, NY, United States; 3 Family Health Centers at NYU Langone, Brooklyn, NY, United States; 4 Division of Preventive and Behavioral Medicine, Department of Population and Quantitative Health Sciences, University of Massachusetts Chan Medical School, Worcester, MA, United States

**Keywords:** health equity, federally qualified health center, hypertension, implementation fidelity, team-based care

## Abstract

**Background:**

Multilevel interventions targeting patients, providers, and health systems improve hypertension outcomes, but little is known about how well they are implemented in resource-limited settings. This study evaluated the implementation fidelity of ALTA, a multilevel approach for hypertension management, supported by practice facilitation, across six primary care practices in a Federally Qualified Health Center (FQHC).

**Methods:**

ALTA included five components delivered by FQHC staff. At the practice level, staff: (1) *identified* medication non-adherence in patients with uncontrolled hypertension and (2) *referred* them to a centralized nurse-led virtual team with a home blood pressure (BP) monitor. The nurse-led team: (3) delivered monthly health *coaching* based on home BP readings; (4) completed *documentation* in the electronic health record (EHR); and (5) *monitored* patients' BP and goals. We used a multi-method approach to assess implementation fidelity (adherence, dose, quality, responsiveness, differentiation) guided by Proctor's Implementation Outcome's Framework. Data sources included a structured EHR-embedded tool, narrative reports, and interviews over 12 months. ALTA adaptations were tracked using the Framework for Reporting Adaptations and Modifications–Expanded.

**Results:**

From 2022–2024, 124 staff across 6 FQHC sites implemented ALTA. Implementation adherence varied across sites. The identify component ranged from 49.0% to 57.9% across sites, while the refer component showed greater variability, ranging from 50.0% to 84.4%. Enrollment into health coaching also varied substantially, ranging from 20.8% to 88.5% across sites. The centralized nurse-led team delivered coaching to 91.4% of enrolled patients, completed all documentation, and conducted monitoring for 94.5%. Median implementation dose (patient exposure) was six coaching visits over 5.5 months, and 2.1 BP readings per week. Implementation quality was high, with comprehensive documentation of coaching visits. Six fidelity-consistent adaptations were made to improve feasibility, including simplifying the adherence screener and adding asynchronous training. Interviews (*n* = 46) highlighted the need for team support, ongoing practice facilitation, and feedback on patients' progress as factors affecting fidelity.

**Conclusions:**

Implementation fidelity varied across the ALTA components and participating sites, with the greatest variability observed in delivery of the site-level components of identify and refer. Centralized components delivered by the nurse-led team (coach, document, monitor) were delivered consistently with high fidelity. Contextual factors like staffing and patient engagement shaped implementation success.

**Clinical Trial Registration:**

ClinicalTrials.gov, NCT03713515.

## Introduction

Hypertension affects 120 million US adults, contributing to one in seven cardiovascular disease deaths and over 500,000 deaths annually (1). Despite well-established guidelines and efficacious treatment, approximately 30%–50% of patients with hypertension remain uncontrolled in primary care (2). The Target:BP initiative of the American Medical Association and American Heart Association has documented improvements in the management of hypertension in practice-based settings (3). This systems-level initiative uses comprehensive, protocol-driven, team-based approaches that involve patients, providers and health systems to improve delivery of high-quality hypertension care. While Target:BP provides a framework for optimizing hypertension control, blood pressure (BP) control remains suboptimal in safety-net practices [e.g., community health centers and federally qualified health centers (FQHC)], which represent an essential network of care for medically underserved and vulnerable populations (4). Recent data shows modest changes in hypertension control among safety-net practices in the US, with less than one-third of practices meeting the Healthy People 2030 goal of a 60.8% control rate (5).

The evidence-to-practice gap in hypertension care in safety-net practices is largely due to two fundamental health system barriers in these settings: (1) suboptimal frequency of clinic visits for rigorous hypertension management and coordination of care and (2) limited capacity to support adequate implementation of initiatives such as Target:BP that require significant practice redesign to accommodate team-based care and/or frequent visits (2, 6–8). Medically underserved populations in safety-net practices face additional unique barriers to engaging with evidence-based initiatives, such as limited health literacy, language discordance, and structural challenges like transportation costs and inability to miss work (9, 10). Consequently, the integration of evidence-based initiatives into safety-net practices has faced multiple barriers, leading to inconsistent implementation (11). If these challenges persist, evidence-based initiatives will remain accessible only to well-resourced practices, while resource-limited safety-net practices struggle to implement them with high fidelity, deepening gaps in hypertension management and outcomes in medically underserved populations (12, 13). This underscores the critical need for research aimed at understanding how well evidence-based multilevel interventions are implemented in safety-net settings, and the contextual factors that shape that process.

Emerging equitable implementation science frameworks (14–18) emphasize that successful implementation of evidence-based interventions is shaped by structural, organizational, and contextual factors that influence whether interventions can be delivered consistently across medically underserved populations and resource-limited settings. These frameworks highlight the importance of implementation strategies that are responsive to the workforce constraints, patient needs and organizational capacity within real-world settings. Consistent with this perspective, we conducted a multi-method evaluation of the implementation fidelity of ALTA (**A**dvancing **L**ong-term Improvements in Hypertension Outcomes through a **T**eam-based Care **A**pproach), a multilevel approach for hypertension management supported by practice facilitation in safety-net settings (19, 20). This implementation evaluation examines how well ALTA was delivered across six real-world primary care settings within a large FQHC and explores contextual factors that contribute to variations in implementation fidelity. A companion paper will report findings from the stepped-wedge cluster randomized controlled trial (RCT) evaluating the clinical effectiveness of ALTA on BP control and medication adherence.

## Materials and methods

### The ALTA intervention

**Figure 1:**
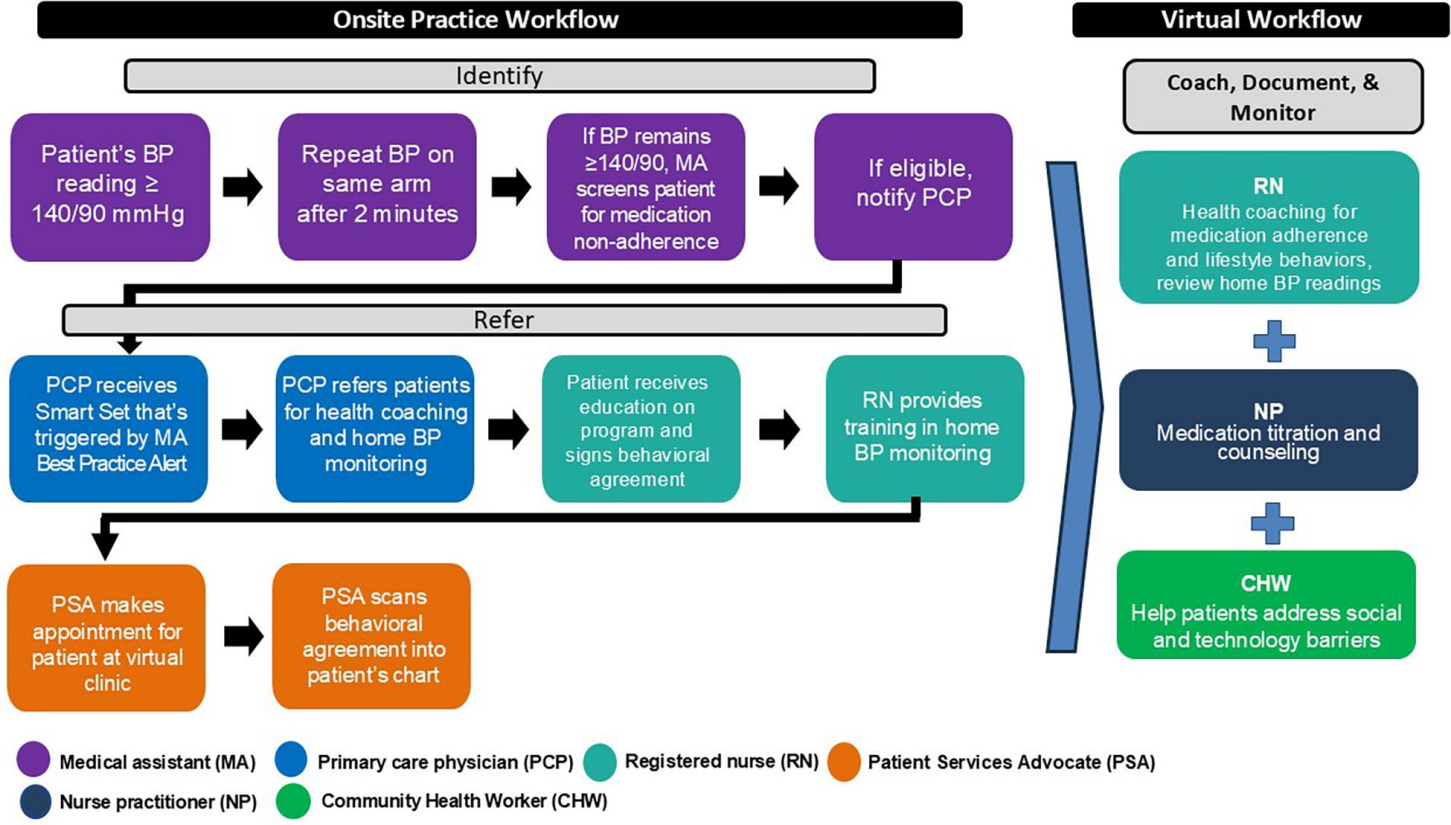
ALTA intervention workflow.

Details on the development of ALTA have been previously reported (19–21). Consistent with principles of community-engaged and patient-centered outcomes research, ALTA was developed and operationalized through ongoing collaboration with the FQHC leadership, frontline staff, and implementation partners to ensure alignment with the needs and constraints of medically underserved populations and safety-net workflows (21). ALTA incorporates the evidenced-based Target: BP protocol to provide practices with a standardized strategy for improved hypertension management using a team-based approach. ALTA has five core components delivered by FQHC staff (implementers, Figure 1). At the local practice level, (1) medical assistants (MA) identify patients with uncontrolled hypertension and medication non-adherence using the electronic health record (EHR); and (2) primary care physicians (PCP) refer eligible patients using clinical decision support (CDS) tools to a centralized nurse-led team of two registered nurses (RN) and a nurse practitioner (NP) and home BP monitoring. The nurse-led team: (3) conducts monthly virtual health coaching visits using a structured tool in the EHR to discuss BP readings and goals; identify barriers and facilitators to medication adherence; develop self-management plans; and use a structured treatment algorithm to optimize the antihypertensive medication regimen; (4) documents progress notes in the EHR to update the care team on the plans and medication changes; and (5) monitors patient BP and schedules follow-up sessions.

During the intervention planning period (prior to any study activities), the research team identified a newly awarded hypertension initiative at the FQHC—*The National Hypertension Control Initiative (NHCI): Addressing Disparities among Racial and Ethnic Minority Populations*- which had significant overlap with ALTA's project goals, target staff and patients, outcomes, and timeline. To create a seamless program, the FQHC leadership and study principal investigator proactively merged the initiatives to create an integrated program with unified messaging and protocols. The integration preserved the core components of the ALTA intervention (Identify, Refer, Coach, Document and Monitor) and their primary functions (Table 1) while adding NHCI-derived components including Bluetooth-enabled remote BP monitoring, patient training on accurate self-BP measurement, community health worker (CHW) support for patients' social and technical needs, and expanded eligibility criteria to include all race and ethnic groups (ALTA originally targeted only Hispanic patients). Patients received a free validated home BP monitor (OMRON BP7250) and were trained to measure their BP twice each morning and evening, with 1 min of rest between readings. They were recommended to do this at least 3 days per week (12 readings in total), with the recommendation of a minimum twice daily for 1 week before the coaching visit. Home BP readings were automatically transmitted via Bluetooth from the patient's phone to the EHR, allowing the virtual nurse-led team to review and discuss them during monthly coaching visits.

**Table 1:** Description of the five ALTA intervention components.

ALTA component	Original core functions	Forms added during planning phase
Identify	Electronic health record (EHR) registry to identify Latino patients with uncontrolled hypertension and who are prescribed antihypertensive medications Screen patients for medication non-adherence using a standardized adherence screening and document the result in the EHR	Broaden criteria to be inclusive of patients of all races and ethnicities
Refer	Clinical decision support tool used by primary care physicians to refer patients to health coaching and recommended use of home BP monitor	Inclusion of referral for Bluetooth-enabled remote BP monitoring and training in self-measured BP
Coach	Structured assessment of medication adherence Use of motivational interviewing to identify facilitators/barriers to adherence and discuss personalized strategies to improve adherence behaviors Use of structured medication titration algorithm to optimize patient treatment regimen Use of teach back to ensure patient understanding of treatment plan Creation of self-management goals and action plans	Coaching delivered by the centralized virtual team Inclusion of CHW to help address social and digital needs
Document	Consistent monitoring of patient progress through completion of and tracking follow-up sessions with ALTA patients	Conducted by a centralized virtual team
Monitor	Structured documentation of coaching sessions in EHR to enable tracking patients' progress over time EHR Documentation of individualized treatment plan, medication adherence goals, and action plan to guide future sessions and share with primary care providers for clinic visits	Conducted by the centralized virtual team

### Usual care phase

Prior to implementation of ALTA, baseline data for usual care over a period of 12 months were extracted from the EHR at each of the six practices. These data included clinic BP readings, prescription refill data (medication adherence defined by the proportion of days covered metric), and practice and patient sociodemographic characteristics (e.g., % of practice population that is female, Hispanic ethnicity, racial groups, mean age, payor mix).

### Pre-implementation phase

Following the usual care phase, practices were block-randomized to receive practice facilitation in three waves, with two sites per wave. Once randomized, sites engaged in an approximate 6–12 month pre-implementation phase, guided by the Consolidated Framework for Implementation Research version 2.0 (22). This time range was due in part to interruptions caused by COVID-related pauses in research activities at the practices. During this phase, trained practice facilitators conducted a multi-method evaluation of three CFIR 2.0 domains: the inner setting, innovation, and individual using a combination of semi-structured interviews, validated surveys, workflow analysis, and environmental scan (19, 23). All practice staff were also required to complete online training in guideline-concordant office BP measurement based on American Heart Association recommendations (i.e., two BP measurements with 1 min of rest between readings), self-measured BP protocols, patient-centered communication, and quality improvement (QI) methods. Results of the pre-implementation phase have been previously published (23). The evaluation findings were presented to an Implementation Committee, composed of practice leaders, providers, staff and research team members from across the practices (21). These activities were central to our participatory implementation process in which FQHC leadership and implementors informed ongoing tailoring of the practice facilitation strategies to the local practice environment, culture, and climate, workflow adaptations, and implementation priorities. They also helped to develop proactive solutions to address practice and patient-level barriers to implementing ALTA.

## Implementation phase

After the pre-implementation phase, practices transitioned to the implementation phase and received the tailored practice facilitation strategy. Practice facilitation is a multi-faceted implementation strategy that can help primary care teams overcome challenges to integrate evidence-based multilevel interventions (24). By providing hands-on tailored support, practice facilitators can enhance implementation fidelity by building practice capacity to adapt workflows, train staff, and effectively use health information technology to ensure consistent delivery of multilevel interventions.

The practice facilitation strategies in the implementation phase were informed by the ERIC (Expert Recommendations for Implementing Change) compilation and designed to foster effective team communication and problem solving skills, enhance capacity to use data to drive improvements, establish and share common implementation goals, and develop skills in continuous QI methods, such as root cause analyses and Plan-Do-Study-Act cycles (Table 2) (25). Practice facilitation emphasized bidirectional feedback between frontline staff, FQHC leadership, and the research team, allowing for iterative refinement of implementation strategies in response to practice and patient-level barriers.

**Table 2:** Description of practice facilitation implementation strategy.

ALTA component	Implementation strategy description	ERIC category
Identify	Provide training in proper blood pressure measurement protocol to assist practice staff in identifying ALTA-eligible patients	Conduct ongoing training
	Conduct Plan-Do-Study-Act cycles to assist practices in implementing screening criteria to identify ALTA-eligible patients	Conduct small cyclical tests of change
	Provide technical assistance in the use of EHR and patient portal to identify ALTA-eligible patients	Provide ongoing consultation
	Develop systems to monitor enrollment of ALTA-eligible patients	Develop and organize quality monitoring systems
Refer	Assist practice staff in developing a workflow that supports referral to a health coach	Workflow assessment and testing
	Assist practice staff in creating a system to identify and act on missed opportunities for referral to a health coach	Audit and provide feedback
	Assist practice staff in onboarding ALTA-eligible patients for home blood pressure monitoring	Provide ongoing consultation (Staff facing) Purposely reexamine the implementation (Patient facing)
Coach	Assist practice staff in using patient-centered communication skills	Training
Monitor	Assist practice staff in identifying opportunities for follow-up	Audit and provide feedback
Document	Assist practice staff in allocating resources for quality improvement activities and developing a reporting process	Develop and organize quality monitoring systems

Practice facilitators were members of the research team who had clinical and/or managerial experience in primary care settings. All practice facilitators received training in the five ALTA components and Target:BP protocols, tools for fidelity monitoring and assessment [e.g., Framework for Reporting Adaptations and Modifications-Expanded (FRAME)] (26–28), and human subjects research and data safety. In addition, all facilitators completed online courses on core topics from the Lean Six Sigma curriculum such as QI and change management methods, patient-centered communication (e.g., motivational interviewing), lean principles, coaching, and health information technology optimization.

A core element of the practice facilitation strategy was the bidirectional collaboration between the study practice facilitators and the centralized FQHC QI team (herein referred to as the implementation team). This partnership was essential for effectively transferring and implementing the intervention core functions from the research team to the FQHC QI team to promote high fidelity in their delivery. Details of the implementation team activities have been previously published (21). Briefly, the implementation team established a collaborative governance structure to jointly manage project implementation while balancing the dual priorities of research rigor of a RCT and real-world clinical practice operations. Roles and responsibilities were clearly defined, with implementation progress tracked via regular EHR audits, shared problem-solving, and iterative feedback to frontline staff and FQHC leadership. Weekly meetings facilitated monitoring of implementation metrics, barrier identification, and co-development of workflow supports such as job aids and targeted action plans. The implementation team also participated in staff huddles to set and align enrollment goals and partnered with FQHC staff on root cause analyses and Plan-Do-Study-Act cycles to refine implementation strategies, addressing any challenges, and adapting strategies to optimize implementation. The implementation team met with the practice providers and staff biweekly in the first 3 months of implementation. Over the remaining 9 months, the implementation team held monthly onsite meetings with additional communication by phone or email, as needed.

## Study sample

The six primary care practices are part of a large FQHC that provides care in Brooklyn, New York capturing a diverse population of mostly Hispanic and Black immigrants (70%) facing poverty, as well as cultural and language barriers (43% are non-English speakers, 72% live at or near poverty level). Most patients (64.2%) at the practices are enrolled in Medicaid or Medicaid Managed Care, while 9.2% are uninsured. Prior to the initiation of ALTA at the practices, the network-wide BP control rate for the FQHC was 68.4% with a range of 62.2%–71.5% across the participating sites.

Practice and patient eligibility criteria were previously described (19). Briefly, practices had to be using the EHR for at least 6 months, be willing to sign a Memorandum of Understanding, and identify a practice champion to join the project implementation team. As a system-wide strategy, all patients aged ≥18 years were eligible to participate in ALTA if they had: (1) a routine primary care appointment in the past 12 months; (2) a hypertension diagnosis (using ICD-10 coding); (3) active in the hypertension registry; (4) uncontrolled hypertension defined as an average office BP ≥ 140/90 mmHg after two readings taken 1–2 min apart at the clinic visit, (5) an active patient portal; (6) active antihypertensive medication(s); and (7) a proportion of days covered score <80% [medication non-adherent]. Patients were ineligible if they: (1) planned to discontinue care at the practice within the next 6 months; (2) their provider deemed them ineligible (e.g., due to a diagnosis of dementia); or (3) currently pregnant or planning to become pregnant within a year. This study was approved by the NYU Langone Health Institutional Review Board (reference number: s18-01290).

## Measures

Consistent with implementation science methodology and Proctor's Implementation Outcomes Framework (IOF), this study evaluated implementation fidelity at the individual implementer level (29). We defined fidelity using five dimensions: (1) adherence to the ALTA intervention protocol by the implementers (clinic providers and staff), (2) dose of ALTA delivered to patients, (3) quality of health coaching tasks delivered by implementers, (4) implementer responsiveness (i.e., satisfaction) to ALTA, and (5) ALTA differentiation as compared to other practice hypertension programs. We used a multi-method approach to capture each dimension of fidelity that combined data extracted from a structured tool embedded in the EHR, narrative reports completed by the implementation team, and semi-structure exit interviews with implementers (19). The primary objective of this evaluation was to characterize *how* well ALTA was implemented rather than to estimate its causal effect on blood pressure control.

The structured tool embedded in the EHR was designed by the investigators based on our pilot work (30) and used checkboxes, active buttons, smart phrases, and structured note taking fields to provide a systematic way for the care team to document information on patients' progress throughout the intervention. The tool also served as a method to assess implementation adherence (i.e., documentation of task completion) and quality. The implementation team used narrative reports to document fidelity to the implementation process at site visits, including any unplanned deviations to the protocol and adaptations to the intervention components. During site visits, the implementation team shadowed the care teams as patients moved through the ALTA workflow, gathered feedback through informal interviews, and conducted root cause analyses or Plan-Do-Study-Act cycles with implementers to improve adherence to the ALTA protocol. At the end of the implementation phase, the research team conducted semi-structured interviews to assess implementers' experiences and satisfaction with ALTA.

The FRAME was used to systematically document and categorize all adaptations identified through structured monitoring by the implementation team (26, 28). Adaptations and their effects on implementation fidelity were assessed using the same multi-method approach employed to assess fidelity throughout the study. Specifically, EHR-extracted data were reviewed at weekly implementation meetings to assess whether adaptations achieved their intended effect of improving feasibility without compromising core intervention functions. Adaptations were classified as fidelity-consistent only after monitoring confirmed that core functions were maintained, as determined by consensus among the implementation team and guided by the FRAME criteria. Unintended effects, such as workflow disruptions or downstream impacts on patient engagement were assessed through narrative reports.

### Implementation fidelity dimensions

We quantified each dimension of implementation fidelity using summary statistics (e.g., mean, percentage) based on a review of methods employed by other implementation studies (31–36), data from the authors' previous studies (30, 37), and discussion among the Implementation Committee about feasibility of measurement. Below and in Table 3, we describe the methods to assess each fidelity dimension and the data sources informing the assessment.

**Table 3:** Domains of implementation fidelity and methods of assessment for ALTA (intervention).

Domain	Goal	Method of assessment	Measure calculation
Adherence	To measure the extent to which implementers adhered to the ALTA intervention protocol, as intended	Data extracted from a structured tool built into the EHR for each ALTA intervention component: MA completed the medication adherence screener PCP placed RPM order and referred patient for health coaching and home blood pressure monitoring Delivery of health coaching visits by the virtual nurse-led clinic Documentation and monitoring patient progress Monthly narrative reports completed by the implementation team that summarized use of the ALTA components, and any adaptations made to the components using the FRAME.	Adherence was rated as a Yes/No metric for each intervention component and further categorized as: Little to no evidence of implementation, as per protocol (implemented ≤33% of the time) Partial implementation (implemented 34%–67% of the time) Mostly implemented and/or modified with permission (implemented ≥68% of the time)
Dose	To measure the extent to which patients were exposed to the ALTA intervention (specifically within the virtual nurse-led clinic)	Patient exposure to ALTA intervention components: Health coaching sessions with the nurse-led clinic Submission of home BP readings	Dose was calculated as mean number (standard deviation) and duration (in months) of patient attendance at the health coaching visits and submission of home BP readings. A*verage program dose was* calculated as the total number of health coaching sessions attended by patients during the study divided by the total number of sessions scheduled. The number of observed vs. expected home BP readings was also calculated.
Quality	To measure the quality of implementers’ delivery of health coaching tasks	Data extracted from a structured EHR tool to document the comprehensiveness of health coaching tasks including use of the structured data fields and free text notes on: Adherence barriers and facilitators Patient-centered goals Self-management plans Summaries with next steps	Quality was calculated as the level of completeness of the tool: Poor (little to no documentation using the tool and/or notes) Acceptable (documentation limited to structured tool without any free text notes) Comprehensive (use of structured data fields with additional free text notes)
Responsiveness	To measure level of staff satisfaction with and acceptance of ALTA	Semi-structured interviews at 12 months with implementers across the sites to assess satisfaction with ALTA and acceptability of the practice changes.	Responsiveness was analyzed using a stepwise deductive and inductive coding approach by three trained coders
Differentiation	To measure the unique features of ALTA that are distinguishable from other hypertension programs at the sites	Monthly narrative reports completed by the implementation team to document all QI-related initiatives that occurred at the practices during the study, the goals of the initiatives, the specific components, the staff involved, and the target patient populations.	Differentiation was calculated as the degree of overlap between the ALTA components and other QI initiatives at the sites: No overlap (unrelated goals, audience, outcomes and/or separate timeline) Some overlap (similar patients and staff targeted but different project goals) Significant overlap (same project goals, timeline, staff and patients targeted)

Implementation adherence was evaluated as the degree to which the five components of the ALTA intervention were implemented by the implementers, as intended (e.g., *identifying* patients with uncontrolled hypertension and screening for medication non-adherence; *referring* patients for nurse-led health coaching and home BP monitoring; providing health *coaching* including reviewing home BP readings and medication adherence, lifestyle coaching, and goal setting; *documenting* and *monitoring* patient progress) using notes from the narrative reports completed by the implementation team, and data extracted from the structured tool in the EHR. Each intervention component (identify, refer, coach, document, and monitor) was first classified as implemented or not implemented (yes/no) and then categorized as follows: little to no evidence the component was implemented, as per protocol (≤33% of the time); the component was partially implemented (34%–67% of the time); and the component was mostly implemented and/or they were implemented with an adaptation (as tracked by the FRAME) that did not affect the intervention's core components (implemented ≥68% of the time) (31).

Implementation dose was evaluated based on the extent of patient exposure to the coaching and monitoring intervention components, using data extracted from the EHR. We extracted data on the number of: (1) patient health coaching sessions with the nurse-led team, and (2) home BP readings uploaded by patients.

Implementation quality was evaluated based on the comprehensiveness of health coaching tasks delivered by implementers, using data extracted from the EHR structured tool. The tasks included: (1) assessing patient barriers and motivation for treatment adherence; (2) identifying a patient-centered goal; (3) creating a self-management plan; and (4) summarizing the session with the next steps. Implementation quality was rated as poor (little to no documentation); acceptable (documentation limited to use of structured data fields only); comprehensive (use of structured data fields with additional free text notes) based on how thoroughly the data for each task were captured in the tool.

Implementer responsiveness was evaluated as practice staff and provider satisfaction with and acceptability of ALTA using semi-structured qualitative interviews conducted at the end of the implementation phase across the practices. Interviews were conducted using a semi-structured guide informed by findings from our pre-implementation evaluation (23) and Proctor's IOF (29).

Program differentiation was evaluated as the unique features of ALTA that were distinguishable from other hypertension programs at the sites. Throughout the study, the implementation team captured data on all hypertension-related programs that occurred at the practices using a structured form including program name, goals, target audience, expected outcomes, and timeline. This data was used to quantify the degree of overlap between the ALTA components and other programs at the practices as well as to isolate the unique features of ALTA that distinguish it from those programs. Overlap was defined as none (unrelated goals, audience, outcomes, and/or separate timeline), some (similar patients and staff targeted but different project goals), and significant (same project goals, timeline, staff and patients targeted).

## Analysis

Implementer and practice characteristics (e.g., payor mix, racial groups, % of patients who are Hispanic ethnicity) were calculated as frequencies and proportions for categorical variables; means and standard deviations (SD) were calculated for continuous variables. Implementation adherence was calculated as the percentage of components mostly, partially, or not implemented, with a range: 0%–100%. We assessed implementation adherence at the implementer level within each practice, as well as across all participating practices. Implementation dose was calculated as the median number (interquartile range) and duration (in months) of the health coaching sessions attended by patients. We also calculated the median (interquartile range) of the observed vs. expected number of home BP monitor readings uploaded to the EHR. Implementation quality was calculated as the percentage of health coaching tasks thoroughly covered across the total number of sessions delivered. Implementation responsiveness was analyzed thematically from semi-structured interviews using a step-by-step coding process, combining deductive and inductive coding by three trained coders. Program differentiation was examined descriptively as the number of hypertension programs with significant overlap with ALTA, compared to those with none or some overlap.

## Results

### Practice characteristics and study population

We enrolled six primary care practices from December 2019 to January 2022. The first of three waves of practices began the pre-implementation phase in December 2020 while the final wave began in January 2023. The year-long implementation phase for wave 1 began in January 2022 and the final wave began September 2023. Among patients with hypertension across all six practices (*N* = 7,154), 56.7% were female, 59.5% identified their ethnicity as Hispanic, and about one-third (29.2%) identified as Black/African American. The mean age of the population was 61.9 (SD: 13.6) years. About half (44.4%) had Medicaid and one-fifth (19.9%) were self-pay. The most common comorbid conditions were obesity (16.9%) and diabetes (15%). A total of 124 implementers (of 176 eligible) across the six practices provided sociodemographic information (Table 4). About one quarter (22.6%) were under the age of 35 years, most were female (83.1%), and one third identified their ethnicity as Hispanic (34.7%). An equal percentage of implementers reported their roles as a PCP (25.8%), MA (24.2%), or other support staff (25.8%) while fewer identified as RNs (9.7%). Nearly half of the implementers (48.4%) had been in their current FQHC position for under 3 years.

**Table 4:** Sample characteristics of the implementers across six practices.

Socio-demographics^a^	All	Site 1	Site 2	Site 3	Site 4	Site 5	Site 6
	(*N* = 124)	(*N* = 21)	(*N* = 10)	(*N* = 26)	(*N* = 20)	(*N* = 35)	(*N* = 12)
	%	%	%	%	%	%	%
Age in years							
<35	22.6	19.1	20.0	15.4	10.0	42.9	8.3
35–45	33.1	28.6	60.0	38.5	45.0	28.6	0.0^b^
46–55	25.8	38.1	20.0	26.9	35.0	14.3	25.0
>55	18.6	14.3	0.0^b^	19.2	10.0	14.3	66.7
Gender							
Male	16.9	9.5	30.0	7.7	10.0	28.6	16.7
Female	83.1	90.5	70.0	92.3	90.0	71.4	83.3
Race and ethnicity							
Black Or African American	14.5	57.1	0.0^b^	15.4	0.0^b^	5.7	0.0^b^
Asian	15.3	0.0^b^	10.0	3.9	0.0^b^	25.7	66.7
Latino/a	34.7	9.5	60.0	50.0	50.0	28.6	16.7
Other	35.5	33.3	30.0	30.8	50.0	40.0	16.7
Born							
USA	54.8	38.1	70.0	57.7	60.0	62.9	33.3
Another Country	45.2	61.9	30.0	42.3	40.0	37.1	66.7
Employment							
Role^c^							
Leadership	14.5	23.8	30.0	11.5	15.0	8.6	8.3
PCP	25.8	14.3	30.0	11.5	20.0	45.7	25.0
RN	9.7	4.8	0.0^b^	11.5	15.0	11.4	8.3
MA	24.2	23.8	0.0^b^	30.8	25.0	17.1	50.0
Other (E.G., PSA)	25.8	33.3	40.0	34.6	25.0	17.1	8.3
Years in position							
0–2	48.4	61.9	50.0	50.0	40.0	57.1	8.3
3–5	15.3	9.5	40.0	15.4	20.0	11.4	8.3
6–15	21.0	14.3	10.0	19.2	15.0	25.7	41.7
16+	14.5	14.3	0.0^b^	15.4	20.0	5.7	41.7

a Column percent is reported overall and by site, for those who responded to the survey. Columns may not sum to 100% due to missingness.

b Note that some cells may contain “0.0” value if no survey *respondent* represented that category.

c PCP, primary care physician (including physicians, physicians assistants, nurse practitioners, and licensed practical nurses), RN, registered nurse, MA, medical assistant, and PSA, patient services advocate.

During the 2-year implementation period (January 2022–December 2023), a total of 3,103 patients met the initial eligibility criteria for participation in ALTA (i.e., hypertension diagnosis, active patient portal and on hypertension registry, repeat clinic BP readings ≥140/90 mmHg in the same encounter, pharmacy refill score <80%). The following section presents the results of the implementation fidelity metrics, which tracks the flow of patients across the five ALTA components.

Table 5 shows the results for implementation adherence for each component, both at the practice level and overall. Across the six practices, MAs partially implemented the *identify* component, with 53.1% (*n* = 1,648) of potentially eligible patients screened for medication non-adherence. PCPs *mostly* implemented the refer component with 72.8% of eligible patients (*n* = 1,200) referred to the nurse-led virtual clinic for health coaching and home BP monitoring via the CDS tool. About fifteen percent (15.7%, *n* = 188) of referred patients (*n* = 1,200) declined participation, 12.8% (*n* = 154) were deemed ineligible by the PCP (e.g., due to cognitive impairments), and 21.3% (*n* = 256) did not complete enrollment for virtual health coaching. Reasons for not completing enrollment included the local practice nurses being unable to complete training in self-measured BP before patients left the site (required before scheduling virtual health coaching visits), patient refusal due to potential co-pays for the virtual clinic visits, or the home BP monitoring order was canceled, which could be patient or provider driven. Thus, a total of 652 (50.2%) patients were scheduled for virtual health coaching. At the practice level, all six sites partially implemented the *identify* component. Half of the sites partially implemented the *refer* component, while the other half mostly implemented it. Analysis of the narrative reports showed that site-level variation in implementation adherence appeared to reflect contextual differences in staffing stability (e.g., turnover of nurse managers who served as practice champions), implementation capacity (e.g., ability to document BP readings in real-time), and patient engagement barriers related to digital literacy and trust.

**Table 5:** Implementation adherence data overall and by site (*N* = 7,154).

Patients with uncontrolled hypertension seen across the six practices from January 27, 2022–September 13, 2024							
ALTA components	Overall	Site 1	Site 2	Site 3	Site 4	Site 5	Site 6^c^
ALTA-eligible patients^a^	3,103 (43.4%)	779 (25.1%)	574 (18.5%)	370 (11.9%)	290 (9.3%)	1041 (33.5%)	49 (1.6%)
MA completed medication adherence screener (identify)	1,648 (53.1%)	451 (57.9%)	319 (55.6%)	212 (57.3%)	131 (45.2%)	512 (49.2%)	24 (49.0%)
PCP placed an order for RPM and referred to nurse onboarding (refer)	1,200 (72.8%)	358 (79.4%)	176 (55.2%)	168 (79.2%)	61 (46.6%)	426 (83.2%)	11 (45.8%)
Patient enrolled in health coaching and home BP monitoring	652 (50.2%)	332 (88.5%)	89 (43.4%)	62 (29.7%)	60 (75.8%)	93 (20.8%)	19 (75.0%)
Patient had ≥1 health coaching session (coach)^a^	596 (91.4%)	318 (95.7%)	82 (92.4%)	51 (80.8%)	48 (80.0%)	83 (88.9%)	14 (76.2%)
Patient had ≥1 structured ALTA progress note (document)^b^	596 (100%)						
Patient had ≥1 follow up session to monitor BP and goals (monitor)^b^	563 (94.5%)						

a Hypertension diagnosis, MyChart active, hypertension registry, PDC < 80%, 2 BP readings ≥140/90 mmHg at clinic visit).

b Centralized task in the nurse-led virtual clinic.

c Smaller number of eligible and enrolled patients due to site's limited patient panel size.

Implementation adherence of the coach, document, and monitor components were centralized within the nurse-led virtual team. The *coach* component was mostly implemented with a majority (91.4%, *n* = 596) of scheduled patients having at least one health coaching visit with a nurse in the virtual clinic. The *document* component was fully implemented with 100% of patients (*n* = 550) having structured documentation of their health coaching visits. The *monitor* component was mostly implemented with 94.5% (*n* = 563) of patients having at least one follow-up visit to monitor their home BP readings and goals.

Data on adaptations tracked by the implementation team using the FRAME are included in the Supplementary File. The implementation team identified six adaptations to ALTA, of which one occurred during the pre-implementation phase and five during the implementation phase. During pre-implementation, the trainings were converted into an asynchronous format with self-paced modules to reduce participation barriers for resource-limited staff with competing clinical demands. During implementation, adaptations, driven primarily by the practice facilitation strategies, focused on the content and context to enhance ALTA's feasibility and adoption. For example, the EHR-embedded medication adherence screener was shortened from five to two items to reduce workflow burden in practices with limited workstations for real-time vital capture and increase MA adoption while preserving its core construct by removing redundant items on skipping or missing doses and combining two items about filling prescriptions. All adaptations were rated as fidelity consistent (i.e., preserved the core functions of ALTA); none were identified that negatively affected implementation or intervention delivery.

The median implementation dose was six health coaching sessions [interquartile range (IQR): 12 (QI = 2, Q3 = 14)] over 5.5 (±5.47) months of participation. During this time, a total of 57,690 home BP readings were uploaded by patients. The median dose for home BP monitoring was 2.1 readings per week with most patients uploading approximately between 1 and 5 BP readings per week [IQR:3.92 (Q1 = 0.73, Q3 = 4.65)], which was lower than the recommended frequency of 12 readings per week. Only 4.5% (*n* = 24) of patients exceeded the recommended frequency of BP readings. Implementation quality was rated as comprehensive, with the centralized nurse-led team showing high competence in completing tasks. Nurses consistently used the structured tool during the health coaching visits, as evidenced by completion of the dropdown menus, checkboxes, and smart phrases documenting patient progress in ALTA. All patients had documentation of an adherence score, adherence barriers, and self-management plans using the structured data fields. Additionally, most patients (90%) had structured notes detailing their motivations for joining the program, understanding of hypertension and BP readings, current lifestyle behaviors, and patient-centered goals.

Implementer responsiveness was evaluated via 32 individual interviews with implementers and four role-based focus groups (*n* = 14 implementers). Analysis identified five primary themes related to satisfaction with and acceptability of ALTA: (a) perceived appropriateness of ALTA for patients influenced acceptability among implementers. The free home BP monitor and nurse-led virtual team were viewed as assets to the intervention while factors such as cost and patient age lowered acceptability. (b) Feasibility of implementation shaped perceived appropriateness. Feasibility perceptions were shaped by two key factors: (1) the expected time to enroll a patient during a clinic visit and (2) the patient's ability to engage meaningfully with the nurse-led virtual team. (c) Complex interventions such as ALTA required ongoing practice facilitation and teamwork. External facilitator assistance combined with team support, improved the perceived feasibility and subsequent fidelity of the ALTA protocol. (d) Over time, implementers recognized ALTA's integration into routine practice. Thus, the lower implementation adherence at some sites (Table 5) likely reflects structural and operational constraints rather than low commitment to the intervention purpose and delivery. Overall, ALTA's integration into practice involved a learning curve that was influenced by contextual factors such as staff turnover, competing clinical demands, workflow integration challenges, and varying levels of digital readiness among staff and patients. For some practices, implementation stabilized overtime often due to strong team collaboration and higher patient enrollment, which increased proficiency with the intervention protocol, leading to its integration into routine practice and the development of a shared mental model. Other practices continued to experience struggles because lower patient enrollment limited intervention exposure, driven by a combination of patient ineligibility, patient declinations, and higher staff turnover, which prevented workflow cohesion. (e) Communicating progress of ALTA was key to maintaining implementer buy-in. Regular updates on practice- and system-level hypertension control improvements were recommended by implementers to show how individual contributions shape patient outcomes. Two other key considerations emerged from this analysis. First, implementers saw potential for scaling ALTA beyond hypertension to conditions like diabetes. Second, concerns about program cost highlighted the need for financial feasibility to ensure long-term sustainability and expansion.

A summary of the program differentiation results is shown in Table 6. The implementation team identified two hypertension programs launched around the same time as ALTA; however, neither program had overlapping implementation timeframes.

**Table 6:** Program differentiation of ALTA compared to other hypertension initiatives at the practices.

Program	Goal	Target	Timeline	Impact
Pharmacy outreach (Practice QI Project)	Incorporated a clinical pharmacist into one clinic's workflow to optimize medication management and improve blood pressure, diabetes control, and statin therapy use.	Uncontrolled hypertension: BP ≥ 140/90 mmHg Uncontrolled diabetes: A1c ≥ 9% Candidate for statin therapy: patients with diabetes and/or cardiovascular disease	October 2021–May 2022	Piloted in 1 practice before the ALTA pre-implementation phase 76 patients engaged 93% of patients had at least 1 medication therapy opportunity 56% of patients achieved BP ≤ 140/90 within 2 months of outreach
BETTER-BP (NIH/NHLBI R01)	Pragmatic clinical trial of a lottery incentive program to promote antihypertensive adherence	Hypertension diagnosis Prescribed ≥1 antihypertensive medication Self-reported medication nonadherence At least 1 clinic systolic BP ≥ 140 mmHg within the prior year	October 2023–July 2024	Recruitment for this trial began after the completion of ALTA at the practice that served as a recruitment site for BETTER-BP

## Discussion

Despite strong evidence that multilevel interventions that target patients, providers and health systems improve hypertension management and outcomes (38), their implementation in safety-net primary care settings remains limited, where resource constraints and structural challenges can pose challenges for high-quality, consistent delivery (39). In this context, understanding whether an intervention was delivered as intended and why fidelity varied is essential for interpreting clinical effectiveness, particularly in resource-limited settings where implementation conditions are rarely ideal. Policy provisions expanding reimbursement for coordinated care, including team-based health coaching and home BP monitoring, have increased the need for evidence on how to implement these approaches with high fidelity in real-world practice (40). This study presents a multi-method evaluation of the implementation fidelity of ALTA, a multilevel approach for hypertension management supported by practice facilitation. A key strength was the assessment of all five dimensions of implementation fidelity defined by Proctor's IOF, providing a comprehensive yet pragmatic evaluation of how implementation varied across practices and implementers.

At the local practices, medical assistants only partially implemented the *identify* component, largely due to workflow integration challenges. This likely led to fewer eligible patients being identified for participation in the intervention. Alternatively, PCPs mostly implemented the *refer* component. We previously demonstrated high uptake of the CDS tool for hypertension referrals by PCPs, which was driven by its efficient, dynamic design that facilitated its use and helped close care gaps (41). The *coaching*, *documenting* and *monitoring* components, which were centralized within the nurse-led virtual team were mostly implemented. Similarly, implementation quality of the coaching sessions was consistently high across the nurse-led team. We attribute the high implementation fidelity of the nurse-led components to several advantages of centralized delivery. These include dedicated staffing and protected time for BP data management and patient outreach, clearer role definition, and reduced workflow variability. In contrast, decentralized practice staff often have more variable responsibilities and face greater time constraints, which may have limited consistency in implementation. However, analysis of our narrative reports suggests that variations in implementation adherence should not be interpreted solely as differences in the practice staff's motivation or performance across the sites. Instead, our findings suggest that structural conditions within safety-net settings (staffing turnover, implementation capacity, digital readiness) shape the ability of practices and staff to fully engage with evidence-based interventions. From an equitable implementation perspective, these contextual factors represent important determinants of whether multilevel, digitally enabled interventions can achieve consistent delivery, reach, and engagement across safety-net settings serving medically underserved populations.

On average, implementation dose met program expectations, with patients attending one health coaching session per month. However, the frequency of home BP readings was below recommendations, with most patients transmitting readings twice per week instead of twice per day. Regarding responsiveness, implementers found ALTA acceptable when it was considered appropriate for patients, with benefits like free home BP monitors and the nurse-led health coaching increasing acceptance. Feasibility depended on the time needed for enrollment and patients' ability to engage with home BP monitoring and virtual visits. Successful implementation required teamwork and support, which improved feasibility and fidelity. Practice facilitation allowed for program adaptations that supported all levels of implementation fidelity, most notably implementation adherence and implementer responsiveness. Importantly, many of the adaptations aimed to preserve engagement among frontline staff and patients who may be at higher risk of implementation failure due to structural and organizational constraints. Viewed through an equity lens, these adaptations were designed to not only improve feasibility, but to also support more consistent delivery of ALTA across practices operating with varying staffing, time, and infrastructure constraints. Over time, some implementers developed a collaborative understanding of ALTA, which facilitated its integration into routine workflows, while others struggled due to staff turnover. Regular updates on patients' progress toward BP control were recommended to help maintain implementer engagement and implementation success. Finally, a coexisting hypertension initiative was proactively integrated into the ALTA components prior to the study launch, without compromising, and potentially enhancing, implementation fidelity.

Our findings are consistent with previous studies that have assessed implementation fidelity using multi-method measurement approaches. For example, Serhal et al., applied Proctor's IOF to evaluate implementation success of the ECHO Ontario Mental Health project (31). While the study showed an overall high level of success, they also found variation over time in key implementation outcomes such as adoption (decrease in patient uptake), feasibility (differences in practitioner participation by role type), and penetration (uneven reach across local health integration networks) at the patient and practitioner levels. Similarly, Sanchez et al., utilized Proctor's fidelity dimensions (adherence, dose, quality of delivery, responsiveness and program differentiation) in a mixed methods evaluation of the PVS-PREDIAPS implementation strategy (42). The strategy was designed to support the adoption of an interprofessional community of practice to optimize primary prevention of type 2 diabetes in primary care. While overall fidelity was high, this study also documented variations across the fidelity dimensions due to differences at the practitioner (e.g., physicians receiving a lower dose compared to nurses) and practice (e.g., lower fidelity of training delivery due to staff turnover) levels (42). A comprehensive analysis of how differences in contextual factors at the site-level including staffing, organizational culture, and practice readiness contributed to this variation is the focus of a forthcoming companion paper. Preliminary findings from implementer interviews suggest that staff turnover, team cohesion, digital health literacy, and needs for practice facilitation support were key factors differentiating higher- from lower-fidelity sites. In retrospect, several strategies may have reduced variation in fidelity across sites, including more intensive and sustained practice facilitation support at lower-performing sites, earlier identification of staff turnover as a fidelity risk with proactive contingency planning, and the need to anticipate and address digital barriers that influence implementation success at the practice, staff, and patient levels. A forthcoming companion paper will provide a comprehensive, realist-informed analysis of the contextual factors driving site-level variation and offer targeted recommendations for optimizing implementation consistency in similar resource-limited settings.

There are several strengths of our study that advances previous research. First, an important strength of this work was the research-practice partnership that integrated constituent engagement throughout implementation planning and delivery. As opposed to traditional clinical trials that deploy a fixed intervention, ALTA's implementation strategy relied on continuous engagement with frontline staff, FQHC leadership, and the centralized QI team to tailor workflows, training approaches, and facilitation strategies to the realities of safety-net primary care practices. This participatory approach is consistent with emerging equity-focused implementation frameworks (e.g., IM4Equity, Digital Health Equity-Focused Implementation Research Model) (14–17) emphasizing the importance of aligning implementation strategies with the priorities, organizational capacities, and lived experience of medically underserved communities and settings. Second, it provides one of the first multidimensional evaluations of implementation fidelity for a multilevel intervention in resource-limited settings serving medically underserved populations. By evaluating fidelity at the implementer level, the study offers robust insights into how specific components of a multilevel model are integrated into routine practice. Moreover, tracking program differentiation enabled alignment with an existing hypertension initiative, allowing for seamless integration into clinical workflows without added burden—an advantage that may not have been achievable without intentionally tracking this dimension. Third, this work adds to the growing but still limited evidence base on practice facilitation by demonstrating how tailored, hands-on support can strengthen implementation fidelity. Practice facilitators helped primary care teams adapt workflows, train staff, and effectively use digital health tools, ensuring consistent delivery of the ALTA components and hypertension management protocols. Fourth, the study employed a multi-method approach that used both structured fidelity metrics that were embedded into the EHR and qualitative reports and interviews that allowed us to document the contextual and organizational factors that drove variations in the fidelity dimensions. Embedding fidelity metrics into the EHR also enabled a pragmatic method to continuously monitor the implementation process in real-time without disrupting clinic workflows. This approach overcame limitations of prior studies that relied on subjective measures of fidelity assessed within a limited time point (often after implementation has ended), which can introduce recall bias and social desirability (29). Fifth, this study documented fidelity-consistent adaptations—such as simplified screening tools and asynchronous training—that can improve feasibility of implementing multilevel interventions in resource-limited settings while maintaining core intervention functions. Collectively, these findings demonstrate how a structured, team-based multilevel intervention—when supported by practice facilitation—can be integrated into safety-net primary care settings that serve medically underserved communities.

Despite these strengths, our study had several limitations. First, the lack of standardized measurement tools and universal definitions for fidelity in the implementation science literature may contribute to variability in how our study operationalized the dimensions as compared to others. This could limit the comparability and generalizability of our findings to other studies (43). Moreover, our fidelity definitions were constrained by the data that was available in the EHR, potentially omitting key aspects of fidelity that were not captured in structured fields. However, we sought to overcome this limitation through the addition of qualitative interviews with implementers and narrative reports by the implementation team. Future research should prioritize psychometric validation of standardized fidelity measures. Our study contributes to this effort by offering a practical tool that can be used in pragmatic clinical trials; additional work is needed to establish its validity and broader applicability. Addressing this gap will improve the robustness of fidelity measurement and support the development of tailored strategies to optimize implementation across varied contexts.

This study was also conducted within a FQHC network, and the unique contextual factors (e.g., patient demographics, resource availability, and workforce composition) of these settings may limit applicability to other practice-based settings. The centralized nurse-led model used in ALTA may have benefited from structural advantages (e.g., centralized resources/staffing) that have important implications when scaling multilevel hypertension interventions to new health systems. Additionally, we did not conduct a formal economic evaluation of the costs to implement this model. Thus, the resource implications, including staff time, training requirements, and technology requirements, were not systematically quantified. This limits our ability to fully characterize the feasibility of scaling this model with high fidelity to other resource-limited settings. Importantly, these findings reflect implementation fidelity during an active intervention period and should not be interpreted as evidence of sustainability. Future implementation studies should include comprehensive economic evaluations of implementation, including adaptation decisions, across diverse healthcare settings and organizational structures to inform scalability and sustainability.

## Conclusion

This study used a multi-method approach to assess the implementation fidelity of a multilevel intervention for improved hypertension management in safety-net settings. Implementation adherence varied across the six participating sites, particularly the site-level components of identify and refer, which relied on local staff workflows. This site-level variability underscores the influence of contextual factors, including dedicated resources and staff, ongoing training, strong teamwork and practice facilitation on implementation success. Maximizing the long-term impacts of ALTA will require enhancing patient engagement with the intervention, as well as addressing staff time constraints that can limit delivery within busy primary care settings. Future studies are needed to examine whether fidelity can be maintained beyond active implementation support and over longer implementation periods. Future research should also focus on standardizing multidimensional fidelity measures to better capture the complexities of implementation fidelity in diverse real-world settings. Together, these efforts are essential to developing more effective and sustainable strategies to improve health outcomes through high-fidelity implementation of evidence-based interventions in practice-based settings.

## Data Availability

The raw data supporting the conclusions of this article will be made available by the authors, without undue reservation.
